# High-fat diet-induced atherosclerosis promotes neurodegeneration in the triple transgenic (3 × Tg) mouse model of Alzheimer’s disease associated with chronic platelet activation

**DOI:** 10.1186/s13195-021-00890-9

**Published:** 2021-08-28

**Authors:** Min Wang, Junyan Lv, Xiaoshan Huang, Thomas Wisniewski, Wei Zhang

**Affiliations:** 1 Key Laboratory of Brain Functional Genomics (Ministry of Education and Shanghai), School of Life Sciences, East China Normal University, 3663 North Zhongshan Road, Shanghai, 200062 China; 2 Center for Cognitive Neurology and Departments of Neurology, Pathology and Psychiatry, New York University School of Medicine, Science Building, Rm1017, 435 East 30th Street, New York, NY 10016 USA

**Keywords:** Alzheimer’s disease, Atherosclerosis, Platelet-rich clots, Cerebral amyloid angiopathy, Cerebral blood flow

## Abstract

**Background:**

Epidemiological studies link vascular disease risk factors such as atherosclerosis, hypertension, and diabetes mellitus with Alzheimer’s disease (AD). Whether there are direct links between these conditions to β-amyloid (Aβ) aggregation and tau pathology is uncertain.

**Methods:**

To investigate the possible link between atherosclerosis and AD pathology, we subjected triple transgenic (3 × Tg) AD mice to a high-fat diet (HFD) at 3 months of age, which corresponds to early adulthood in humans.

**Results:**

After 9 months of treatment, HFD-treated 3 × Tg mice exhibited worse memory deficits accompanied by blood hypercoagulation, thrombocytosis, and chronic platelet activation. Procoagulant platelets from HFD-treated 3 × Tg mice actively induced the conversion of soluble Aβ40 into fibrillar Aβ aggregates, associated with increased expression of integrin αIIbβ_3_ and clusterin. At 9 months and older, platelet-associated fibrillar Aβ aggregates were observed to obstruct the cerebral blood vessels in HFD-treated 3 × Tg mice. HFD-treated 3 × Tg mice exhibited a greater cerebral amyloid angiopathy (CAA) burden and increased cerebral vascular permeability, as well as more extensive neuroinflammation, tau hyperphosphorylation, and neuron loss. Disaggregation of preexisting platelet micro-clots with humanized GPIIIa49-66 scFv Ab (A11) significantly reduced platelet-associated fibrillar Aβ aggregates in vitro and improved vascular permeability in vivo.

**Conclusions:**

These findings suggest that a major contribution of atherosclerosis to AD pathology is via its effects on blood coagulation and the formation of platelet-mediated Aβ aggregates that compromise cerebral blood flow and therefore neuronal function. This leads to cognitive decline.

**Supplementary Information:**

The online version contains supplementary material available at 10.1186/s13195-021-00890-9.

## Background

Alzheimer’s disease (AD) is the most common cause of dementia among the elderly, affecting approximately 50 million people currently and with projections being ~150 million affected by 2050 [1, 2]. Currently, there were no effective pharmacological means to treat or slow down this progression. AD is characterized by two dominant pathological hallmarks [2, 3]. One is the abnormal deposition of endogenous β-amyloid (Aβ) peptides (Aβ_1-40_ and Aβ_1-42_) in the brain parenchyma forming senile plaques and in the walls of cerebral vessels producing cerebral amyloid angiopathy (CAA) [4–9]. The other is the intracellular accumulation of the microtubule-associated protein tau in its hyperphosphorylated form resulting in the formations of neurofibrillary tangles (NFT) in neurons.

A substantial body of studies indicates that vascular damage and dysfunction, such as reduction of cerebral blood flow (CBF) and blood-brain barrier (BBB) disturbances, could be one of the earliest events contributing to the onset and progression of AD [10]. Vascular dysregulation has been linked with CAA. The presence of CAA and its severity is an independent factor for dementia [11, 12]. Almost 100% of AD patients have CAA, and in about a third of these patients, it is rated as severe CAA [11, 13]. The presence of CAA promotes the onset of AD symptoms [14] and is associated with faster cognitive decline in non-cognitively impaired (NCI) individuals [14, 15]. The presence of CAA is also associated with tau pathology [11, 16–19]. Hyperphosphorylated tau deposits have been reported to be significantly more likely to be found in areas of the brain affected by CAA [19]. A recent study showed that AD individuals with CAA were more likely to develop severe NFT pathology relative to those without CAA [16]. These findings indicate that the presence of CAA is an important factor influencing the severity of tau-related pathology. Therefore, identifying vascular risk factors that facilitate CAA lesions is potentially useful for the early diagnosis and treatment of AD patients.

Epidemiological studies link atherosclerosis with an increased risk for dementia and AD [20, 21]. However, whether these processes in the vasculature initiate the pathologic process of Aβ aggregation and accelerate tau pathology is still uncertain. Atherosclerosis is a chronic progressive vascular disease and is often accompanied by sustained platelet activation, increased platelet numbers, and the formation of platelet thrombi [22]. Platelets play an important role in CAA pathogenesis, in addition to their fundamental role in arterial thrombosis and hemostasis. Platelets contain high concentrations of amyloid precursor protein (APP) in their alpha granules (~1.1 ± 0.3 μg/10^8^ platelets) and express all of the enzymes which are required to process APP into Aβ peptides. In the human blood, ~90% of Aβ peptides are from platelets [23–27]. Platelets from AD patients showed abnormalities of platelet morphology and APP metabolism [28]. Moreover, platelet-derived Aβ can pass through the human cerebrovascular endothelial cell layers isolated from the brains of patients with AD [29], and these secreted Aβ peptides are similar to those found in amyloid plaques of AD patients [30].

Platelet adhesion under conditions of high shear stress, as occurs in stenotic atherosclerotic arteries, is pivotal to the development of arterial thrombosis. Evidence shows that platelets are 300–500 times more concentrated in blood clots than in non-clotted blood [31]. In this study, we hypothesize that the major contribution of atherosclerosis to AD is via its effects on blood coagulation and chronic formation of platelet micro-clots, which sequester and enrich numerous activated platelets, thus allowing a massive release of Aβ peptides (directly, or cleaved from released APP) and the conversion of soluble Aβ40 into fibrillar Aβ aggregates at the surface of platelet micro-clots. The formation of platelet-associated amyloid aggregates in cerebral vessels may compromise cerebral blood flow and hence neuron survival and function, leading to cognitive decline. We tested this hypothesis in a well-characterized triple transgenic (3 × Tg) mouse model of Alzheimer’s disease, which is one of the few models with both Aβ and tau-related deposits [32, 33]. In this model, Aβ aggregation is found not only in brain parenchyma but also in the cerebral vessel walls [33, 34]. In addition, platelets from this AD model have been documented to be normal in number and glycoprotein expression, but are more adherent to matrices such as fibrillar collagen, von Willebrand factor (vWF), fibrinogen, and fibrillary amyloid peptides compared to platelets from age-matching wild-type (WT) mice [35].

## Methods

### Reagents

All reagents were purchased from Sigma–Aldrich (St. Louis, MO, USA) unless otherwise indicated. Primary antibodies used in this study are listed in Table S1 (Additional file 1). Soluble Aβ (1–40) (Shenggong Co., Ltd., Shanghai) sequence (single-letter code), DAEFRHDSGYEVHHQKLVFFAEDVGSNK GAIIGLMVGGVV was used. Human monoclonal single-chain variable fragment (scFv) antibody (Ab) against platelet GPIIIa49-66 (A11) and control scFv Ab were prepared as previously described [36].

### Animals

3 × Tg mice (human APP KM670/671NL (Swedish), MAPT P301L, and PSEN1 M146 V) exhibiting amyloid and tau pathologies and B6129S control mice were purchased from the Jackson Laboratory (Bar Harbor, ME) and were used to conduct the experiments described. The animals were maintained in an environmentally controlled room at 22° ± 1°C with a 12-h light/dark cycle in a specific pathogen-free facility at the East China Normal University (Shanghai, China). All mice were housed in clear polycarbonate micro-isolator cages (five mice per cage), allowed free access to water and food. Both males and females were included in approximately equal ratios for all experiments. The detailed number, age, and sex of mice used for each experiment are shown in the figure legends. All procedures in the animal experiments were approved by the Institutional Animal Care and Use Committee of East China Normal University. All methods were performed in accordance with the relevant guidelines and regulations.

### Experimental design

#### Experimental animal model of atherosclerosis

There were three experimental groups: 3 × Tg mice on a high-fat diet (HFD), 3 × Tg mice on a normal diet, and B6129S mice on a normal diet. The 3 × Tg mice were randomly separated into two groups. The HFD containing 1.25% cholesterol in order to induce atherosclerosis. The control group 3 × Tg mice were fed normal chow. B6129S control mice were also fed normal chow and served as a comparison group to evaluate if 3 × Tg mice on normal chow have any alterations in hematological parameters and/or vascular permeability. HFD treatment was initiated at 3 months of age, which corresponds to early adulthood in humans. Most mice were fed a HFD for 9 months, and samples were collected at 12 months of age for the subsequent assays. To investigate the initial signs of AD vascular lesions, 2–3 mice in each group were randomly selected and monitored at 6- and 9-month time points. At the end of treatment (12 months of age), animal behavior was analyzed by an observer blinded to the treatment status of the mice. Before assessment of cognitive deficits and locomotor testing, the bodyweight of each mouse was weighted to ensure that any behavioral differences observed in the tasks tested could not be related to differences in body weight (e.g., HFD mice being obese). Serum total cholesterol (TC) was measured with commercial ELISA kits according to the manufacturer’s instructions of the ELISA kit (LYBD Bio-Technique Co., Ltd., Beijing, China). Oil red O staining was used to assess the size of the atherosclerotic lesion and its lipid content. Briefly, mice were sacrificed by the cervical dislocation. Thoracic-abdominal aortas (TAs) were dissected, and oil red O staining of the artery plaque area was performed. For quantification, ImageJ version 1.50i (NIH, Bethesda, MD; http://imagej.nih.gov/ij) was used to measure the lesion size of TAs.

#### In vivo assessment of cerebral blood vessel permeability

In this experiment, 12-month-old 3 × Tg mice fed by HFD or normal chow as well as age- and sex-matched B6129S control mice fed by normal diet were used to assess cerebral blood vessel permeability. B6129S control mice were used to see if 3 × Tg mice alone have alterations in vascular permeability. Cerebral blood vessel permeability assay was performed using Evans Blue dye as previously described [37]. The rationale is as follows: Evans blue is a diazo salt fluorescent dye with high affinity (10:1) for albumin (the most abundant protein in plasma) and presents red fluorescence under the excitation of 550 nm. Under physiologic conditions, the endothelium is impermeable to albumin, so Evans blue bound albumin remains restricted within blood vessels. In pathologic conditions that promote increased vascular permeability endothelial cells partially lose their close contacts and the endothelium becomes permeable to small proteins such as albumin. This condition allows for extravasation of Evans Blue in tissues. Briefly, prepare a 0.5% sterile solution of Evans blue in phosphate buffer saline (PBS) and filter-sterilize the solution to remove any particulate matter that has not dissolved. Evans blue solution (4 ml/kg) was slowly injected through the tail vein of the mouse. Evans blue dye was allowed to circulate for 30 min. Animals were then perfused transcardially with PBS until fluid from the right atrium became colorless. All the mice were sacrificed at the same time, as fast as possible. The brains were harvested immediately, the cerebellum was removed, and the remainder was split in half into two hemispheres. One half of the brains were sliced into 35-μm sections using a cryostat. Tissue sections were thaw-mounted directly onto glass slides and stored at −80°C until use. The level of cerebral vascular permeability can be assessed by simple visualization of the brain section under the excitation of 550 nm by a fluorescence microscope. The other half of the brains was used for quantification of Evans blue extravasated in tissue. Briefly, the brains’ dye was extracted with formamide overnight at 50°C. Subsequently, the brains were allowed to dry for 1 h at room temperature (RT) before being weighed. Formamide dye concentration was quantified spectrophotometrically at 611 nm and normalized to the dry weight of brain hemispheres.

#### In vivo assessment of the effect of A11 on cerebral vascular permeability

A11 is a humanized scFv Ab that preferentially binds to activated platelets and can lyse platelet thrombi [36]. To investigate the effect of A11 on cerebral vascular permeability, 6-month-old HFD-treated 3 × Tg mice were randomly separated into two groups and intraperitoneally injection (*i.p.*) by A11 or control scFv Ab (25 μg/mouse) 2 times every week for 3 months. Then, mouse memory deficits and vascular permeability were analyzed.

### Brain and serum sampling

Mice were sacrificed by the cervical dislocation, and the brain tissue was dissected from mice, weighed, and homogenized in 0.1 M PBS buffer (pH 7.4) containing protease inhibitor cocktail at 1 g/10 mL at 4°C. After centrifugation at 12,000×*g* for 10 min, the supernatant was collected for subsequent biochemical analysis. For serological analysis, mice were deeply anesthetized by *i.p.* injection of pentobarbital (50 mg/kg body weight) and the blood was collected from the retro-orbital sinus. The blood was allowed to clot, then centrifuged at 3000×*g* for 5 min and sera-frozen at −80°C until analysis.

### Sandwich ELISA and Western blotting

The concentrations of serum IL-6 and TPO and the concentrations of reactive oxygen species (ROS), glutathione (GSH), and endostatin (ET) in brain tissues were measured by Sandwich ELISA according to the manufacturer’s instructions of the ELISA kit (LYBD Bio-Technique Co., Ltd., Beijing, China). For Western blotting, proteins were separated on sodium dodecyl sulfate polyacrylamide gel electrophoresis (SDS–PAGE) under reducing conditions and then transferred onto a polyvinylidene difluoride (PVDF) membrane. The membrane was blocked in blocking buffer [PBS, 0.5% Tween-20, and 5% non-fat dry milk powder or 3% bovine serum albumin (BSA)] and then incubated with primary antibody for 1 h at RT. After washing, the membrane was incubated with horseradish peroxidase (HRP)-conjugated secondary antibody for 1 h at RT. The immunoreactive bands were visualized with enhanced chemiluminescence (ECL) Western blot kit (Millipore, Boston, MA, USA) and quantified using ImageJ version 1.50i.

### Quantitative real-time RT-PCR analysis

RNA was extracted from the liver tissue using an RNeasy Mini Kit (Qiagen). The cDNA fragments were reverse-transcribed from mRNA using a high-capacity cDNA reverse transcription kit (Thermo Fisher). Quantitative real-time RT-PCR (*q*RT-PCR) was performed using the Step One Plus real-time PCR system (ThermoFisher, Carlsbad, CA) with SuperReal PreMix Plus (SYBR Green; TIANGEN). The mouse thrombopoietin (*Tpo*) primers were as follows: Forward primer (5′-CCAGGTCCCCAGTCCAAATC-3′) and reverse primer (5′-AATGCCAGGGAGCCTTTGTT-3′). The relative quantity of *Tpo* mRNA was determined using the *ΔΔCt* method, with *Gapdh* as the reference gene. All reactions were performed in triplicates.

### Murine platelet preparation and function testing

Murine blood from retro-orbital plexus was collected and centrifuged at 250 × *g* for 5 min at RT. To obtain platelet-rich plasma (PRP), the supernatant was centrifuged at 50 × *g* for 6 min. PRP was washed twice at 650 × *g* for 5 min at RT, and pellet was resuspended in Tyrode’s buffer (136 mM NaCl, 0.4 mM Na2HPO4, 2.7 mM KCl, 12 mM NaHCO3, 0.1% glucose, 0.35% BSA, pH 7.4) supplemented with prostacyclin (0.5 mM) and apyrase (0.02 U/ml). Before use, platelets were resuspended in the same buffer and incubated at 37°C for 30 min. To determine the bleeding time, the mouse tail vein was severed 2 mm from its tip. Immediately after injury, the tail was placed into a cylinder with isotonic saline at 37°C and bleeding time was measured from the moment the tail was surgically cut until bleeding completely stopped. Platelet counts and mean platelet volume (MPV) were determined by an auto hematology analyzer. The expressions of platelet glycoprotein GPIIb (αIIb or CD41) and GPIIIa (β3 or CD61) were determined by flow cytometry and Western blotting, respectively.

### Murine platelet culture, Congo red staining, and immunofluorescence analysis

Mouse platelets from different treatment groups were cultured in a concentration of 2 × 10^6^ per 100 μl in sterilized glass plate placed in 96-well plate containing DMEM medium and stimulated with 50 μg/ml Aβ40 for 48 h at 37°C. After incubation, unbound platelets were removed by rinsing with PBS, whereas adherent platelets were fixed with 2% paraformaldehyde and stained for fibrillar Aβ aggregates with Congo red according to the manufacturer’s protocol (Merck). Images of fibrillar Aβ aggregates in the platelet cell culture were then photographed by microscope. To determine the effect of A11 on the formation of fibrillar Aβ aggregates in vitro, different concentrations of A11 (0, 10, and 25 μg/ml) and control scFv Ab were simultaneously added to culture systems for 48 h at 37°C and the positively stained fibrillar Aβ aggregates were enumerated under the microscope. For immunofluorescence analysis, the mouse platelet and fibrillar Aβ aggregates in sterilized glass plate were separately stained with anti-GPIbα (rat origin) and anti-Aβ (anti-β-Amyloid, 1-16 antibody, rabbit origin) at 4°C overnight, then incubated with Cy3-labeled (anti-rat) or FITC-labeled (anti-rabbit) secondary antibody (reacted with anti-GPIbα and anti-Aβ, respectively) at RT for 1 h. Images were obtained by Leica SP8 confocal microscope (Leica.Microsystems, Wetzlar, Germany).

### Histology analysis

Mice were deeply anesthetized by *i.p.* injection of pentobarbital and subjected to trans-cardiac perfusion with 0.9% saline buffer followed by 4% paraformaldehyde (PFA) at a slow, consistent rate. The brains were post-fixed overnight in 4% PFA and cryoprotected for 72 h in 30% sucrose solution. The brains were then frozen on powdered dry ice and sliced into 35 μm sections using a microtome. The vascular and parenchymal Aβ deposits in brain tissue sections were visualized with Congo red staining as previously described [38]. In brief, sections were stained with Congo red and images were collected at the selected regions from the frontal cortex to the hippocampus of each mouse brain under the same illumination conditions. Quantification of Congo red staining was performed using the ImageJ software for separately quantifying vascular and parenchymal amyloid deposits in brain tissue sections. For immunofluorescence analysis, brain sections were incubated with anti-Aβ, anti-glial fibrillary acidic protein (GFAP), anti-GPIbα, anti-NeuN, or anti-phospho-Tau396 antibodies. Following three washes of 10 min each with tris-buffered saline (TBS), sections were incubated for 2 h with secondary antibodies conjugated to specific fluorophores for detection. Controls with no primary antibody showed no fluorescence. Samples were counterstained with 4′,6-diamidino-2-phenylindole (DAPI) and imaged with a Leica SP8 confocal microscope. Densitometric analysis of immunofluorescence was performed by using the fluorescence measuring function of ImageJ version 1.50i.

### Contextual fear conditioning test

Contextual fear conditioning was performed to assess associative emotional memory of mice as described previously [39]. This test has been previously used in 3 × Tg mice, demonstrating impaired performance compared to wild-type mice [40]. In the training phase, each mouse was pre-exposed to the shock chamber and allowed to explore the environment for 3 min and a subsequent foot shock (0.5mA) for 2 s. The mice were allowed to stay in the chamber for another 30s, and then, they were placed back into their home cages. The training phase was conducted for 2 days. Approximately 24 h after training, each mouse was placed back into the shock chamber for 3 min during which the freezing behavior of the mouse was recorded (contextual fear conditioning).

### Statistics

Data are shown as mean ± SD. For two independent data comparisons, unpaired *t* test was used to determine statistical significance. For multiple comparisons, one-way ANOVAs were used as indicated in the text. All statistical analyses were performed using the software package Prism version 7 (GraphPad, La Jolla, CA, USA). A *p* value < 0.05 was considered statistically significant.

## Results

### HFD-induced atherosclerosis facilitates memory deficits

**Fig. 1:**
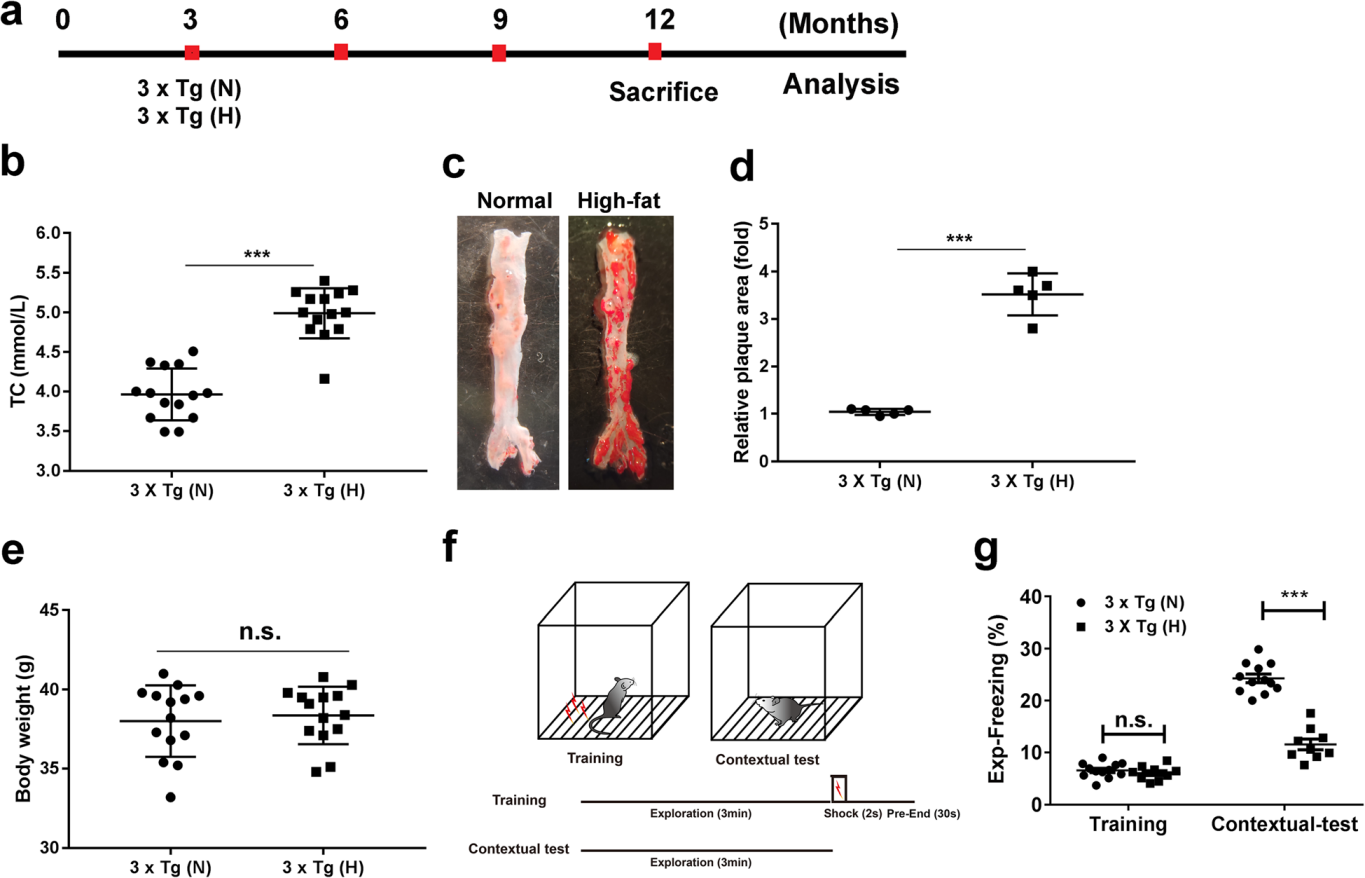
High-fat diet (HFD)-induced atherosclerosis facilitates memory deficits. **a** Treatment protocols to induce atherosclerosis in 3 × Tg mice. N, normal chow; H, High-fat diet. **b** Serum total cholesterol (TC) in 12-month-old 3 × Tg mice after different treatment (*n* = 14/group). **c** Representative images of *en face* oil red O staining of artery plaque area in thoracic-abdominal aorta (TA) of 12-month-old 3 × Tg mice after different treatment. **d** Quantification of the plaque area of TA by ImageJ version 1.50i (*n* = 5/group). **e** Body weight after different treatment (*n* = 14/group). **f** Diagram of methods for hippocampus-dependent contextual fear conditioning. After training, mice were allowed to explore 3 min in shock chamber (Contextual test). **g** Freezing responses of both HFD-treated 3 × Tg mice (*n* = 9) and normal chow-treated mice (*n* = 12) during cued fear conditioning. Each dot or square represents one individual. Data are expressed as mean ± SD, unpaired Student *t* test, two-tailed. ^***^p < 0.001, n.s., not significant

To mimic the chronic pathological progress of atherosclerosis, 3-month-old 3 × Tg mice, which correspond to early adulthood in humans, were fed with HFD for 9 months and were analyzed at 12 months of age (Fig. 1a). After 9 months of treatment, HFD-treated 3 × Tg mice exhibited a significant increase in serum total cholesterol compared to normal chow-treated 3 × Tg mice (Fig. 1b). Oil red O staining showed that the artery plaque area in the thoracic-abdominal aorta of HFD-treated 3 × Tg mice was significantly increased by approximately 3.7-fold in comparison to that of normal chow-treated mice (Fig. 1c, d, respectively). There was no significant difference in body weight between the two groups (Fig. 1e). For cognitive testing, we assessed associative emotional memory through hippocampus-dependent contextual fear conditioning (Fig. 1f). HFD-treated 3 × Tg mice showed significantly decreased contextual fear freezing time (Fig. 1g), suggesting that HFD-induced atherosclerosis facilitates the memory deficits.

### HFD-induced atherosclerosis causes blood hypercoagulation

To determine whether the memory deficits observed in HFD-treated 3 × Tg mice is caused by atherosclerosis-induced circulatory deficits, blood serums from HFD-treated and normal-chow 3 × Tg mice were analyzed by label-free mass spectrometry (MS). A total of 1790 proteins were identified (data not shown). The abundance of 86 proteins (4.8%) was significantly different between these two different cohorts. Gene ontology (GO) term and pathway analyses of significantly changed proteins by Metascape revealed enrichment of proteins of several pathways related to complement and coagulation cascades, blood coagulation, platelet degranulation, and cell-substrate adhesion (Additional file 1, Figure S1). The related molecules include vWF, Complement (C)3, C5, Cfi, Alpha-1-antitrypsin (SERPINA1), Apolipoprotein A-I (APOA1), Inter-alpha-trypsin inhibitor heavy chain H1 (ITIH1), Fibulin-1 (FBLN1), and Gelsolin (GSN) (Additional file 1, TableS2).

### Reduced bleeding time, increased platelet size, and platelet numbers in HFD-treated 3 × Tg mice

**Fig. 2:**
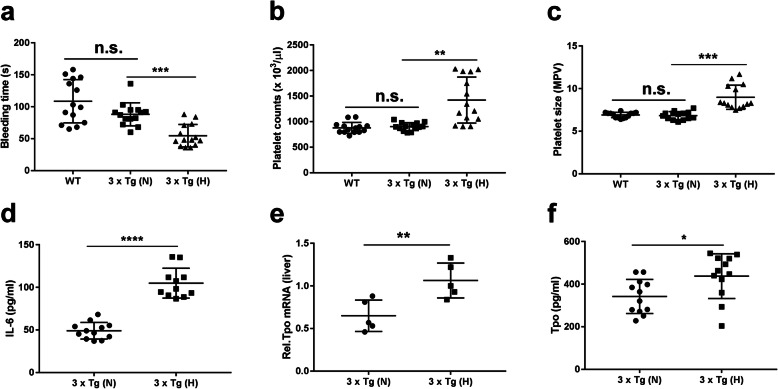
Increased blood coagulation, platelet size, and platelet production in HFD-treated 3 × Tg mice. **a–c** Mouse tail bleeding times (**a**), peripheral blood platelet counts (**b**), and mean platelet volume (MPV) **(c)** in 12-month-old HFD-treated 3 × Tg mice, normal chow-treated 3 × Tg mice, and B6129S wild-type (WT) mice (*n* = 14/group). **d** Serum IL-6 levels in 12-month-old 3 × Tg mice after different treatment (*n* = 11–12/group). **e** *q*RT-PCR analysis of thrombopoietin (*Tpo*) mRNA from the livers of 12-month-old 3 × Tg mice after different treatment (*n* = 5/group). The relative quantity of *Tpo* mRNA was normalized to that of the housekeeping gene *Gapdh* using the *ΔΔCt* method. **f** Serum TPO levels in 12-month-old 3 × Tg mice after different treatment (*n* = 12/group). Each dot or square represents one individual. Data are expressed as mean ± SD, one-way ANOVA (**a**–**c**) or unpaired Student *t* test, two-tailed (**d**–**f**). ^*^p < 0.05, ^**^p < 0.01, ^***^p < 0.001, ^****^p < 0.0001, n.s., not significant

We further assessed the coagulation function in mice. HFD-treated 3 × Tg mice showed a significant reduction in bleeding time and an increase in platelet counts and MPV compared to those of normal chow-treated 3 × Tg mice; these hematological parameters showed no significant differences between normal chow-treated 3 × Tg mice and B6129S WT control mice (Fig. 2a–c, respectively). HFD-treated 3 × Tg mice also exhibited significant elevation of serum IL-6 (Fig. 2d), which is an important pro-inflammatory cytokines stimulating megakaryocytes (MKs) to produce blood platelets [41]. Given that IL-6 could bind with liver IL-6 receptor (IL-6R) and results in increased TPO generation, we examined the levels of TPO. TPO is mainly produced by hepatocytes and interacts with the c-Mpl receptor expressed on megakaryocytic lineage cells in the bone marrow, which contributes to the differentiation of MKs from hematopoietic stem cells and the generation of platelets [42]. The qRT-PCR analysis showed that liver *Tpo* mRNA was 1.4-fold higher in the HFD-treated mice than in the normal chow-treated mice (Fig. 2e). Serum TPO levels also exhibited a significant increase in HFD-treated 3 × Tg mice (Fig. 2f). These data suggest that HFD treatment promotes platelet production (thrombocytosis) associated with elevated IL-6 and TPO levels.

### Platelets from HFD-treated 3 × Tg mice promotes the conversion of soluble Aβ40 into fibrillar Aβ aggregates associated with increased expressions of integrin αIIbβ_3_ and clusterin

**Fig. 3:**
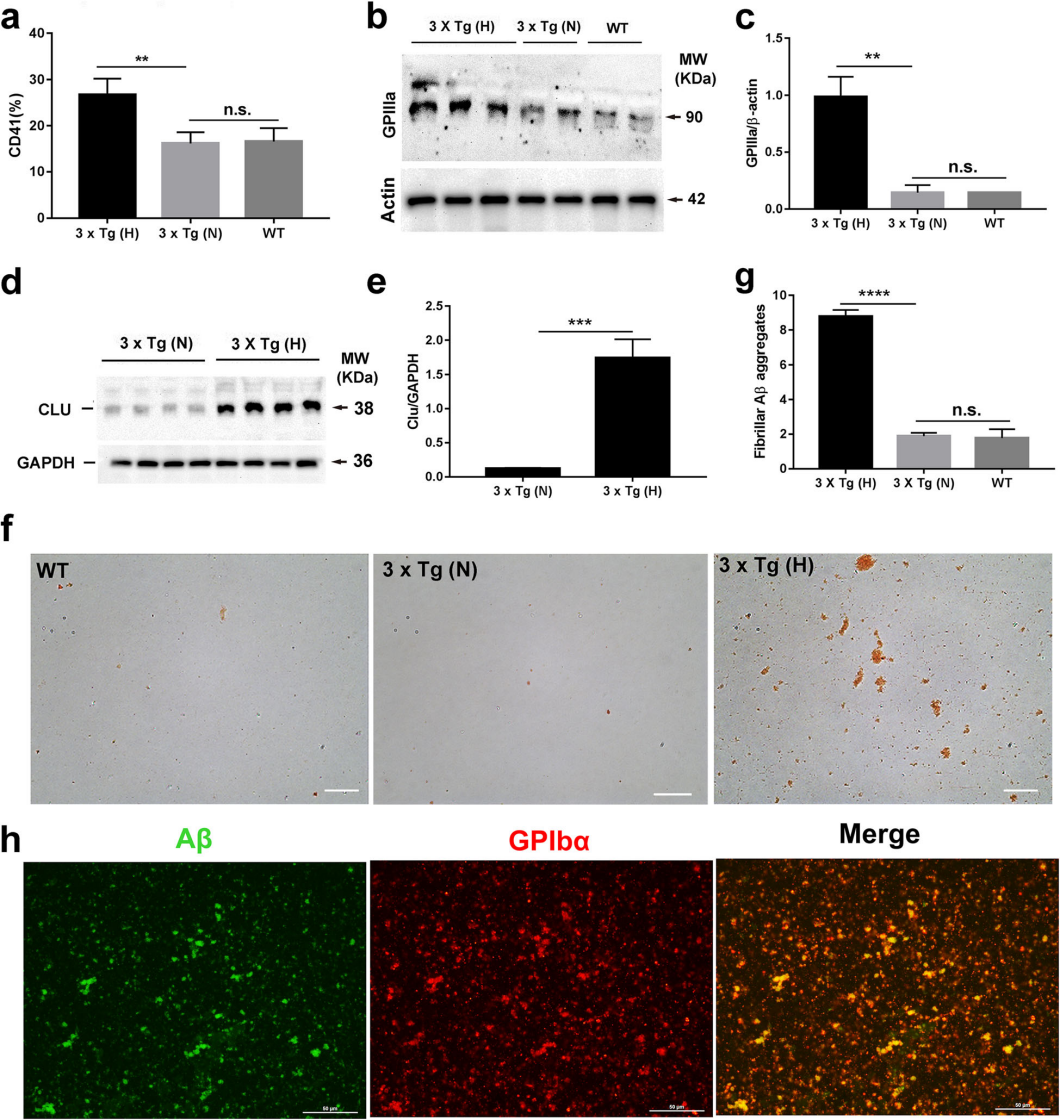
Platelets from HFD-treated 3 × Tg mice actively induce the formations of fibrillar Aβ aggregates in association with increased integrin α_IIb_β_3_ and clusterin (CLU) expression. **a**–**c** The expression of platelet integrin α_IIb_β_3_ determined by flow cytometry (**a**) and Western blot (**b**, **c**) in B6129S wild-type (WT) (*n* = 2), normal chow-treated (*N*) 3 × Tg mice (*n* = 2), and high-fat diet-treated (H) 3 × Tg mice (*n* = 3). **d**, **e** Increased clusterin (CLU) expression in platelets from HFD-treated 3 × Tg mice. Levels of CLU in platelets from 12-month-old 3 × Tg mice after different treatment were determined by Western blotting (**d**). The relative quantity of CLU proteins is normalized to that of GAPDH and expressed as mean ± SD (*n* = 4/group) (**e**). **f** Representative images of Congo red staining-positive Aβ fibril formation in cultures of mouse platelets from different treatment incubated with soluble Aβ40 (50 μg/ml) for 48 h at 37°C. Scale bar, 50 μm. **g** Quantification of the number of fibrillar Aβ aggregates (*n* = 3/group). **h** Analysis of Aβ fibrils adhere to platelet from HFD-treated 3 × Tg mice by immunofluorescence staining. Platelets (red, staining with anti-GPIba); Aβ (green, staining with anti-Aβ1-16). Scale bar, 50 μm. Data are expressed as mean ± SD, one-way ANOVA (**a**, **c**, **g**) or unpaired Student *t* test, two-tailed (**e**). ^**^p < 0.01, ^***^p < 0.001, ^****^p < 0.0001, n.s., not significant. These experiments were repeated independently at least three times

Integrin αIIbβ3 was used as a classical platelet activation marker. Higher baseline expression levels of integrin αIIbβ3 have been previously detected in AD patients with a more rapid cognitive decline compared to patients with a slower decline [43]. The results of flow cytometry and Western blotting showed that the expression of integrin αIIb (GPIIb or CD41) and β3 (GPIIIa or CD61) was significantly increased in platelets from HFD-treated 3 × Tg mice compared to those in platelets from normal chow-treated 3 × Tg mice. However, their expressions showed no significant difference between normal chow-treated 3 × Tg mice and B6129S WT control mice (Fig. 3a–c, respectively). Clusterin is generated by activated platelets upon binding of Aβ40 to platelet integrin αIIbβ3 and contributes to fibrillar Aβ aggregation in the cerebral vessels [44]. Western blotting showed that platelets from HFD-treated 3 × Tg mice contained higher levels of clusterin than those from normal chow-treated 3 × Tg mice (Fig. 3d, e, respectively). In cultures of platelets from HFD-treated 3 × Tg mice, in which αIIbβ3 expression was higher than that of control, the formations of Congo red staining-positive fibrillar Aβ aggregates were apparent after 48 h in culture with Aβ40. In contrast, the formation of Aβ fibrils was barely observed in cultures of mouse platelets from normal chow-treated 3 × Tg mice and B6129S WT control mice under the same conditions (Fig. 3f, g). By immunofluorescence staining, Aβ fibrils (Aβ, green) were found to adhere to platelets from HFD-treated 3 × Tg mice (GPIbα, red) and formed micro-clots (yellow) (Fig. 3h), which supports the hypothesis that soluble Aβ40 is converted into fibrillar Aβ aggregates at the surface of platelet micro-clots.

### Increased CAA burden, formation of platelet-associated fibrillar Aβ aggregates, and oxidative stress in cerebral vessels of HFD-treated 3 × Tg mice

**Fig. 4:**
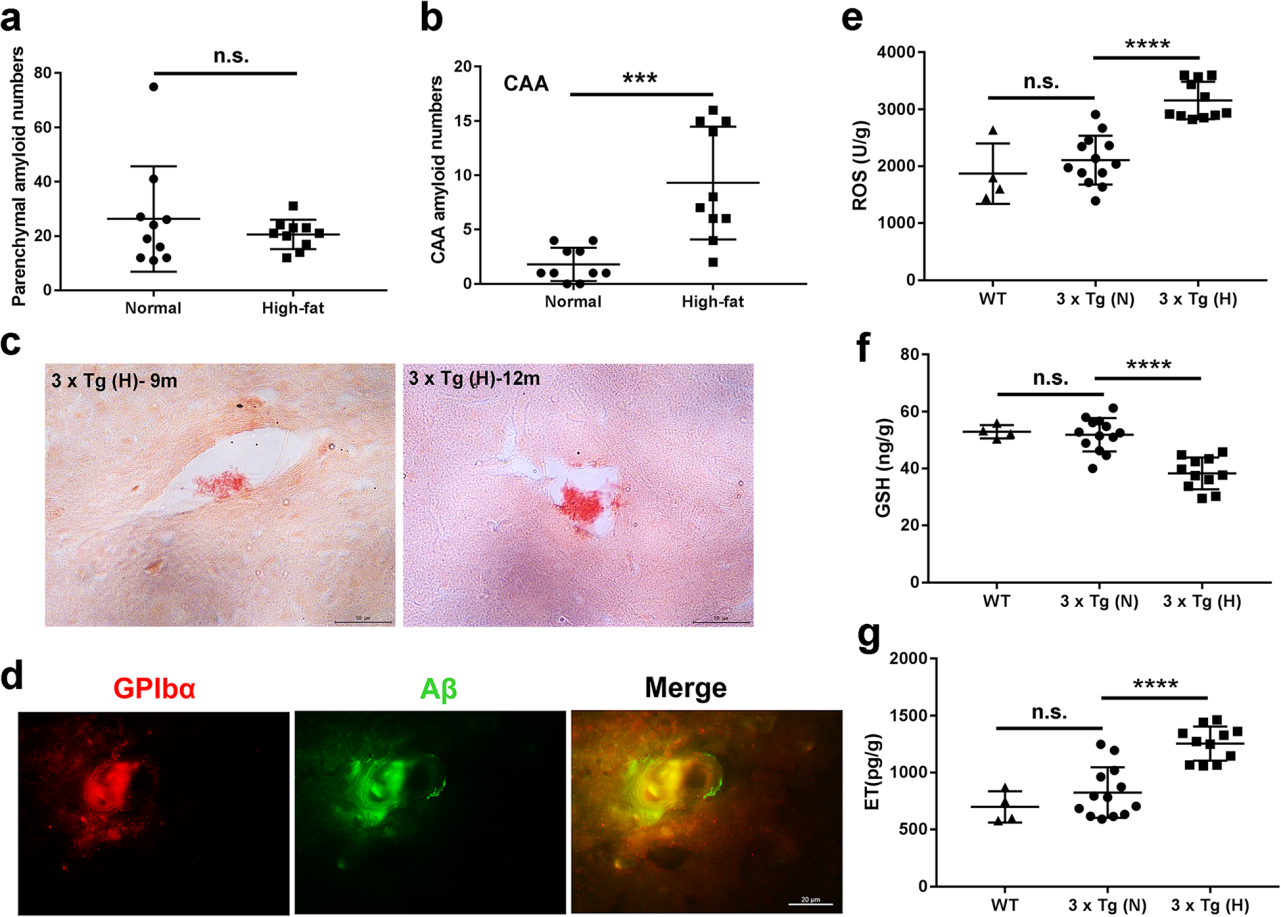
Increased CAA burden, formation of platelet-associated fibrillar Aβ aggregates, and oxidative stress in the cerebral vessels of HFD-treated 3 × Tg mice. **a**, **b** Quantification of parenchymal amyloid numbers (**a**) and CAA amyloid numbers (**b**) using Congo red stainings. **c** Representative images of Congo red staining-positive micro-clots in different sized cerebrovascular vessels of HFD-treated 3 × Tg mice at 9 months and older. Scale bar, 50 μm. **d** Representative immunofluorescence images of the colocalization of platelet thrombi and vascular Aβ plaques in the cerebral vessels of HFD-treated 3 × Tg mice. Overlay of Aβ immunofluorescence (green) and GPIbα (red) is shown in yellow (merge). Scale bar, 20 μm. **e**–**g** The levels of reactive oxygen species (ROS) (**e**), glutathione (GSH) (**f**), and endostatin (ET) (**g**) in mouse brain tissues were determined by sandwich ELISA. Each dot or square represents one individual. Data are expressed as mean ± SD, unpaired Student *t* test, two-tailed (**a**, **b**) or one-way ANOVA (**e**–**g**). ^***^p < 0.001, ^****^p < 0.0001; n.s., not significant

At 6 months of age, mice in the different treatment groups were randomly selected to detect possible initial AD vascular lesions. HFD-treated 3 × Tg mice (3 of 3 mice) were found to have the first sign of CAA lesions in their vascular walls (Additional file 1, Figure S2). However, CAA lesions were barely detected in normal chow-treated 3 × Tg mice (3 of 3 mice) at this age (data not shown). At 12 months of age, Congo red staining was performed, with separate quantification of vascular and parenchymal amyloid deposits in brain tissue sections. Although parenchymal Aβ numbers were comparable between the two groups (Fig. 4a), HFD-treated 3 × Tg mice exhibited a significantly increased CAA burden compared to normal chow-treated 3 × Tg mice (*p* < 0.001) (Fig. 4b). We also found Congo red stain-positive micro-clots in different sized cerebrovascular vessels of HFD-treated 3 × Tg mice at 9 months and older (Fig. 4c). These were barely observed in the vascular lumen of normal chow-treated 3 × Tg mice, even at 12 months of age (data not shown). Immunofluorescence analysis showed that in the vascular lumen of 12-month-old HFD-treated 3 × Tg mice platelet micro-clots (GPIbα, red) adhere to vascular Aβ deposits (Aβ, green) leading to vessel occlusion (yellow fluorescence) (Fig. 4d). At 12 months of age, the levels of ROS were significantly increased and GSH was decreased in the brain tissues of HFD-treated 3 × Tg mice compared to those in normal chow-treated 3 × Tg mice (*p* < 0.001) (Fig. 4e, f, respectively). Given that endostatin is secreted by pericytes upon ROS stimulation resulting in the contraction of the cerebral capillaries [45], we then examined its levels in the brain tissue of different treatment groups. The results showed that the levels of endostatin were significantly increased in the brain tissues of HFD-treated 3 × Tg mice compared to those in normal chow-treated 3 × Tg mice (*p* < 0.001) (Fig. 4g). The levels of ROS, GSH, and ET showed no significant differences between normal chow-treated 3 × Tg mice and B6129S WT control mice at the age of 12 months of old (Fig. 4e–g).

### Increased cerebral vascular permeability and aggravated neuroinflammation in HFD-treated 3 × Tg mice

**Fig. 5:**
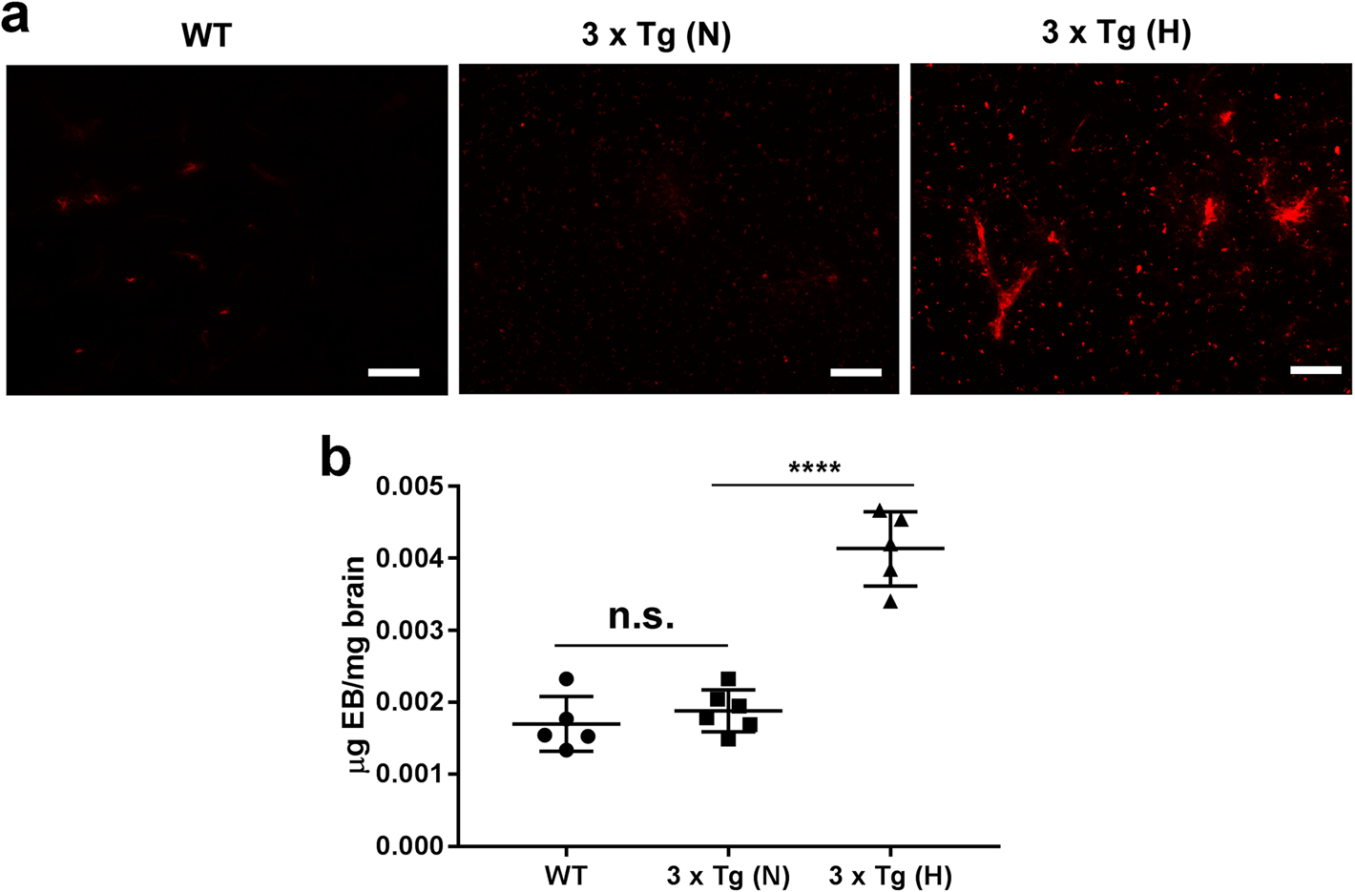
Increased cerebral vascular permeability in HFD-treated 3 × Tg mice. **a** Representative images showing cerebral vascular permeability through simple visualization of brain sections under the excitation of 550 nm by fluorescence microscope. Scale bar, 20 μm. Evans blue is a diazo salts fluorescent dye with high affinity for albumin, and Evans blue bound albumin presents red fluorescence under the excitation of 550 nm. **b** Quantification of Evans blue extravasated in brain tissues as described in the methods. Each symbol represents one individual (*n* =5/group). Data are expressed as mean ± SD, one-way ANOVA. ^****^p < 0.0001, n.s., not significant

**Fig. 6:**
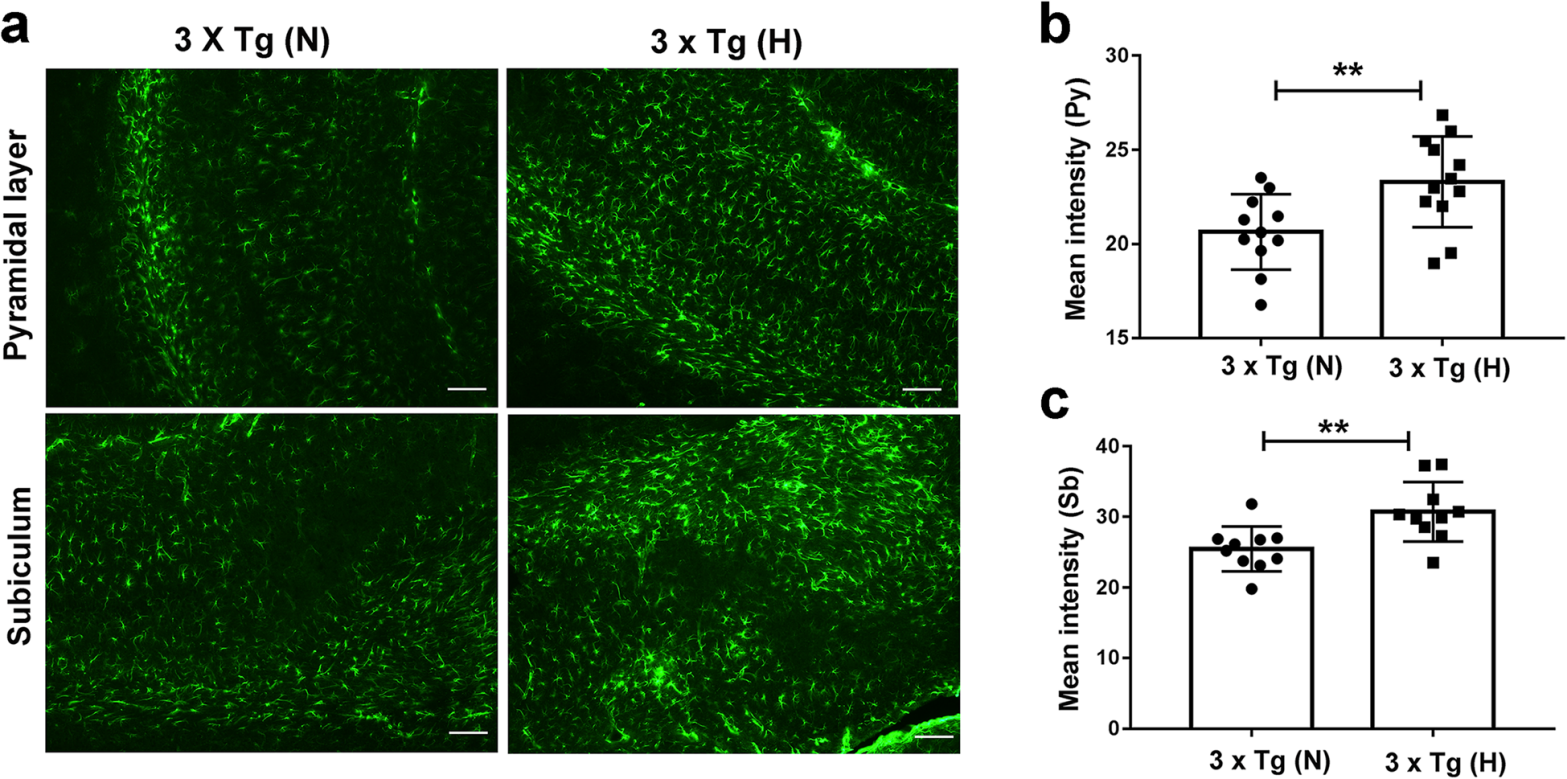
Increased neuroinflammation in HFD-treated 3 × Tg mice. **a** Representative images of glial fibrillary acidic protein (GFAP) immunoreactivity in pyramidal (Py) layer and subiculum (Sb) of mouse brains. **b**, **c** Quantification of GFAP intensity in Py layer (**b**) and Sb (**c**) of mouse brains (*n* =10~12/group). Each dot or square represents one individual. Data are expressed as mean ± SD, unpaired Student *t* test, two-tailed. ^**^p < 0.01

Changes in cerebral vascular permeability were also assessed by Evans blue dye extravasation as described in the methods [37]. Under the excitation of 550 nm, there was a marked increase in red fluorescence in the cortex of HFD-treated 3 × Tg mice compared to normal chow-treated 3 × Tg mice and B6129S WT control mice (Fig. 5a). The content of Evans blue extravasated per milligram of the tissue also showed significantly increased in the brain tissues of HFD-treated 3 × Tg mice (Fig. 5b), suggesting that blood-brain barrier integrity in HFD-treated 3 × Tg mice was compromised. There were no significant differences in vascular permeability between normal chow-treated 3 × Tg mice and B6129S WT control mice. Given the increased vascular permeability in HFD-treated 3 × Tg mice, we then examined neuroinflammation in these mice. GFAP(+) cells were significantly increased in the pyramidal layer (py) and subiculum of HFD-treated 3 × Tg mice (Fig. 6a–c, ^**^p < 0.01), suggesting an increased neuroinflammatory response.

### Increased tau pathology and loss of neurons in HFD-treated 3 × Tg mice

**Fig. 7:**
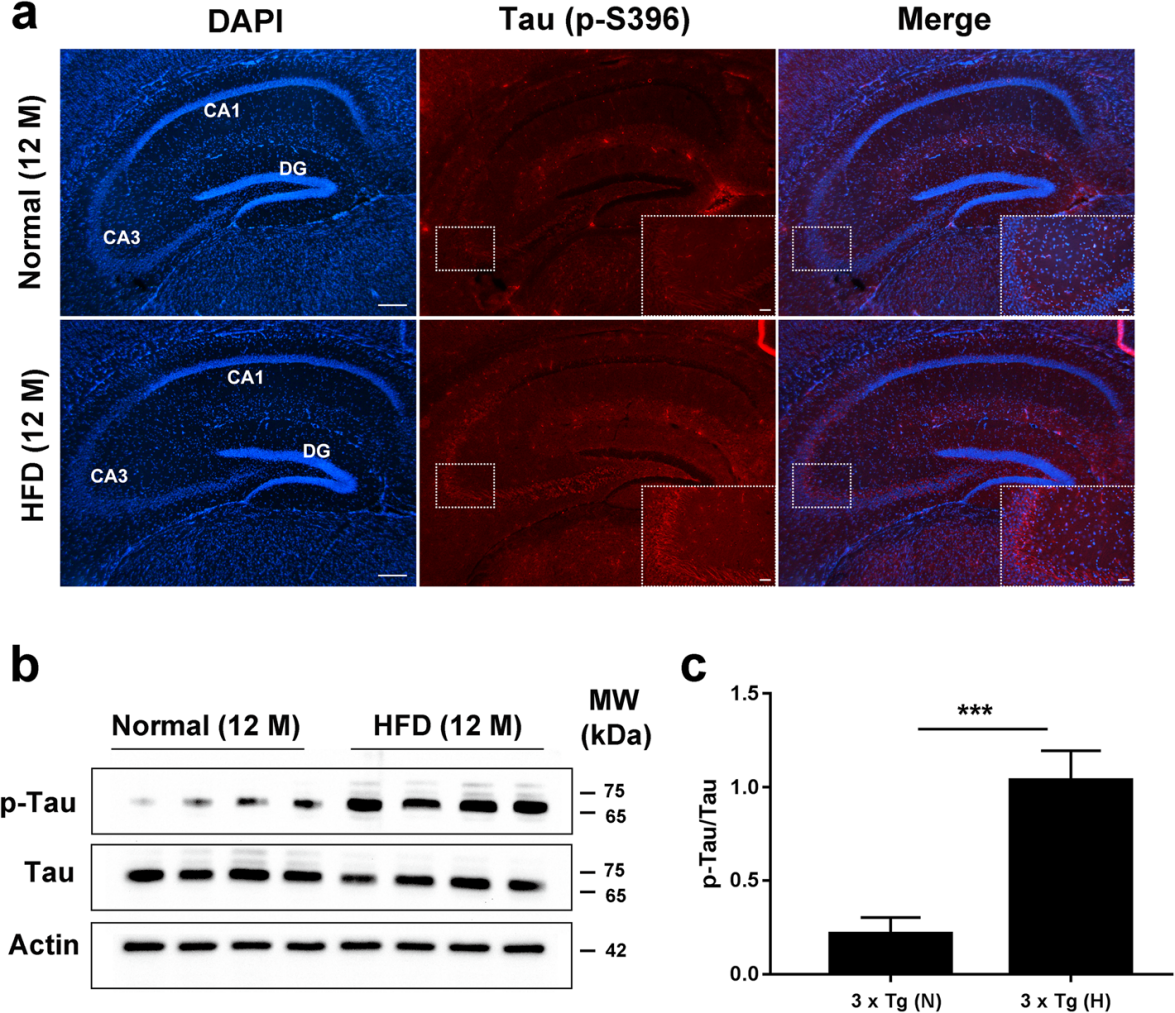
Increased tau pathology in HFD-treated 3 × Tg mice. **a** Representative immunohistochemical images of tau hyperphosphorylation (p-S396) in different hippocampal subregions (CA1, CA3, and DG). Scale bar, 200 μm (overall pictures) and 50 μm (enlarged pictures in the lower right corner of the overall pictures), respectively. **b** Western blotting results of Tau and p-Tau in the brain tissues of different-treated 3 × Tg mice. **c** The relative quantity of p-Tau proteins is normalized to that of Tau and expressed as mean ± SD (*n* = 4), unpaired Student *t* test, two-tailed. ^***^p < 0.001

**Fig. 8:**
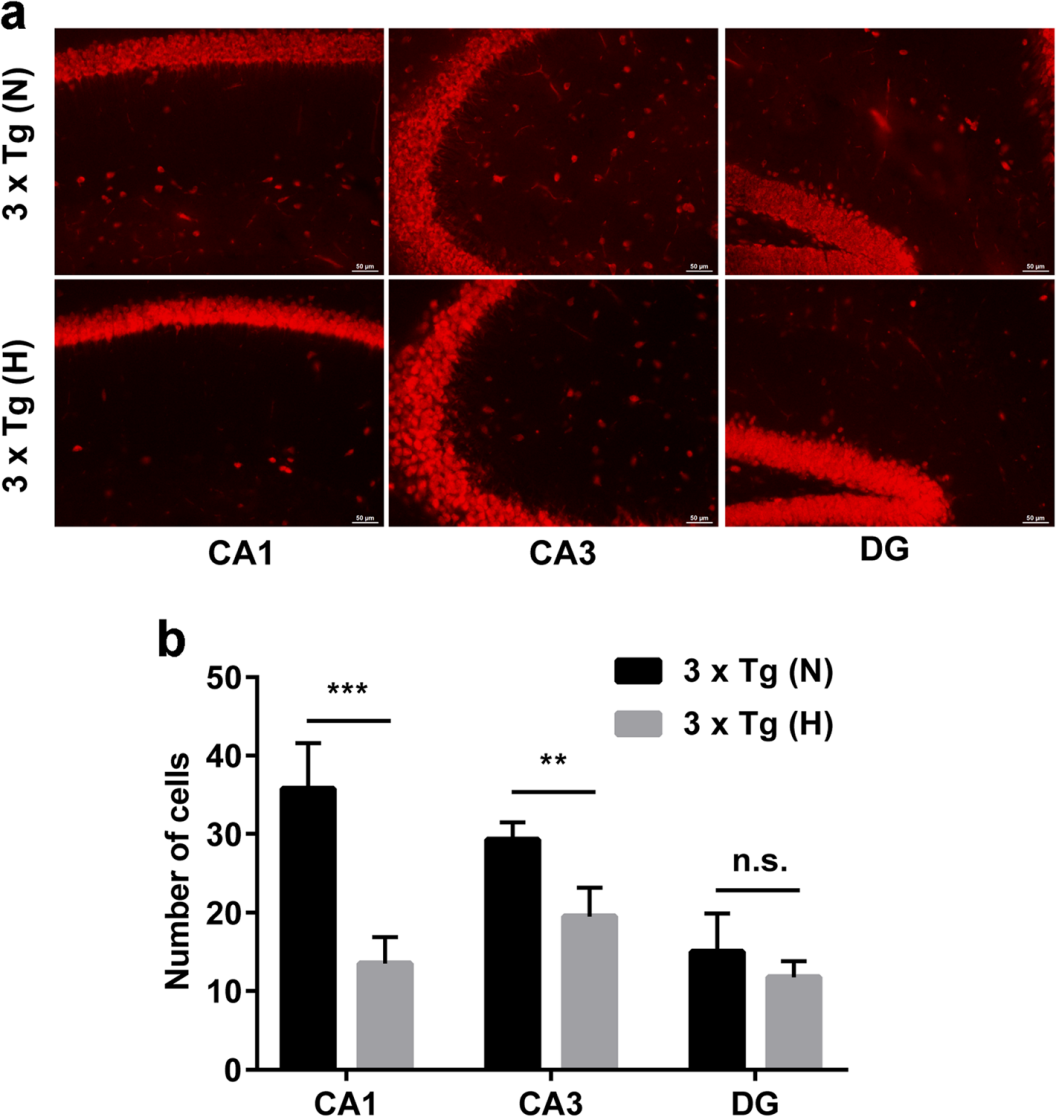
Increased loss of neurons in the hippocampus of HFD-treated 3 × Tg mice. **a** Representative immunohistochemical images of neurons at different hippocampal subregions stained with anti-NeuN antibody. Scale bar, 50 μm. **b** Quantification of the numbers of NeuN-positive neurons at different hippocampal subregions (*n* = 3/group). Data are expressed as mean ± SD, unpaired Student *t* test, two-tailed. ^**^p < 0.01; ^***^p < 0.001; n.s., not significant

Given that CAA, oxidative stress, and inflammation have been proposed as additive variables contributing to promoting NFT pathology [46], we thus examine tau pathology in HFD-treated 3 × Tg mice. Tau pathology typically starts at ~12 months of age in 3 × Tg mice [33]. Normal chow-treated 3 × Tg mice at the end of the experiment (12 months of age) have limited tau hyperphosphorylation in different hippocampal subregions (Fig. 7a). However, HFD-treated 3 × Tg mice have much more extensive tau hyperphosphorylation in hippocampal regions at 12 months of age. Western blotting confirmed that the levels of p-Tau were significantly higher in HFD-treated 3 × Tg mice than in the brain of normal chow-treated 3 × Tg mice (Fig. 7b, c, respectively). Given the compromised vascular system in HFD-treated 3 × Tg mice, we also examined hippocampal sub-regions for evidence of increased neuronal death related to hypoperfusion. NeuN immunohistochemical staining showed that the neuron numbers in the hippocampal CA1 (more prone to cell loss with hypoxia) and CA3 regions were significantly decreased in 12-month-old HFD-treated 3 × Tg mice compared to those in normal chow-treated 3 × Tg mice (*p* < 0.01) (Fig. 8a, b).

### The effect of A11 on cerebral vascular permeability

**Fig. 9:**
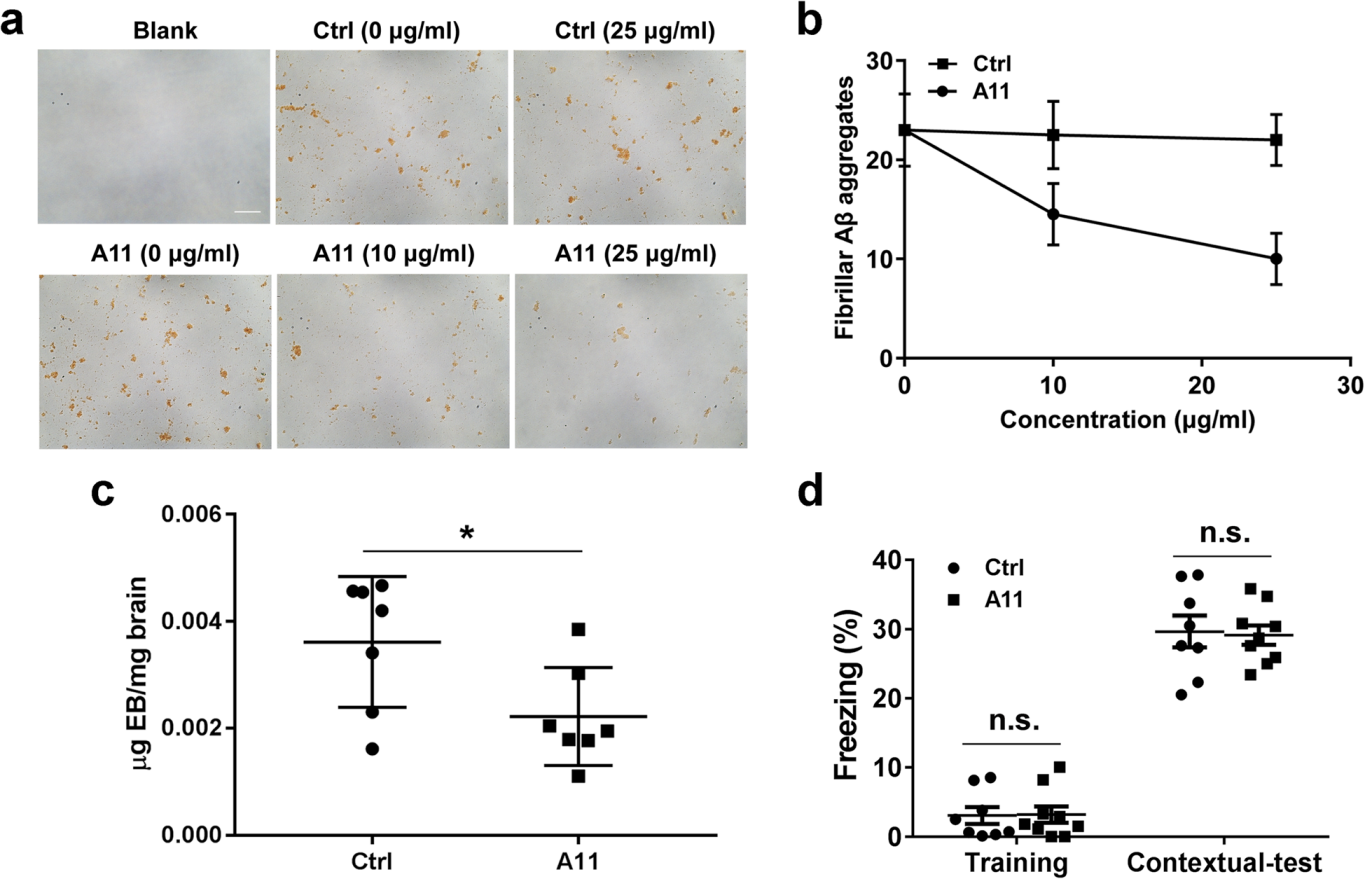
Effect of humanized anti–GPIIIa49-66 scFv Ab (A11) on cerebral vascular permeability. **a** Representative photomicrograph showing that A11dose-dependently inhibited fibrillar Aβ aggregate formation in the cultures of platelets from HFD-treated 3 × Tg mice in culture with Aβ40 compared to irrelevant control scFv Ab. Scale bar, 20 μm. **b** Quantification of the numbers of fibrillar Aβ aggregates (*n* = 4 culture wells/dose). **c** Quantification of Evans blue extravasated in the brain tissues of different groups. Each dot or square represents one individual. **d** Freezing responses of both A11 and control scFv Ab treatment during cued fear conditioning. Each dot or square represents one individual (*n* = 8–9/group). Data are expressed as mean ± SD, unpaired Student *t* test, two-tailed. ^*^p < 0.05; n.s., not significant

A11 is a humanized scFv Ab that preferentially binds to activated platelets and can lyse platelet thrombi [36]. In vitro, A11 dose-dependently inhibited fibrillar Aβ aggregate formation in the cultures of platelets from HFD-treated 3 × Tg mice in culture with Aβ40 compared to irrelevant control scFv Ab (Fig. 9a, b). In vivo, 6-month-old HFD-treated 3 × Tg mice were treated by A11 or control scFv Ab two times every week for 3 months. Although tau-related pathology is limited at this age, A11 treatment did improve vascular permeability (Fig. 9c). A11-treated mice had no noticeable changes in fur, body weight, appetite, spontaneous bleeding, or life span. No significant pathological changes were observed in the brain, heart, liver, kidney, or lung by histologic examination (Additional file 1, Figure S3), suggesting that the treatment was apparently harmless to the mice. However, a contextual fear memory was not improved by A11 treatment (Fig. 9d).

## Discussion

Understanding how co-concurrent disease states contribute to AD is important for early diagnosis and the development of therapies for AD patients. In this study, we fed 3 × Tg mice with HFD and demonstrated that HFD is capable of eliciting the formation of platelet-associated fibrillar Aβ aggregates, increased CAA burden, tau pathology, and loss of neurons. The ideal study design should include both 3 × Tg mice on normal chow and 3 × Tg mice on HFD, alongside WT mice on normal chow and WT mice on HFD, to allow for a more full understanding of what changes are due to the interaction of 3 × Tg and HFD. In this study, we did not include a group of WT mice on HFD, since many of the platelet measures in these mice on HFD have been well documented. Renato et al. showed that platelets from HFD-treated C57BL6/N mice were larger and hyperactive and presented oxidative stress when compared to control C57BL6/N mice on a standard laboratory diet, possibly due to alterations in platelet generation or higher platelet turnover [47]. Santosh et al. found that HFD in B6SJL mice amplified surface P-selectin expression on platelets and increased aggregation of platelets induced by thrombin [48]. Nagy et al. reported that platelets are hyper-reactive in HFD-treated C57BL6 mice, which was partially due to the activation of the adenosine diphosphate (ADP) receptor P2Y12-mediated pathway [49]. Similarly, our data demonstrated that platelets from HFD-treated 3 × Tg mice were increased in size and number and had elevated glycoprotein αIIbβ3 expression. The data suggests that HFD induces platelet hyperactivity in different mouse strains, contributing to hypercoagulability. Given that HFD-treated WT mice do not develop Aβ plaques or tau pathology, HFD-treated 3 × Tg mice are more appropriate to study the contributions of vascular factors to AD-related pathology.

Extensive data indicates that vascular factors play an important role in the pathogenesis of AD. The AD brain has altered blood flow [50, 51] and impaired vascular function [17]. In addition, increased levels of prothrombin [52], thrombin [53], and platelet activation [54, 55] were detected in AD patients. Furthermore, cerebral emboli have been detected in patients with AD and are associated with cognitive decline [56]. In this study, we found that blood coagulation was significantly activated in the blood of HFD-treated 3 × Tg mice associated with molecules, such as Vwf, Fbln1, and prothrombin (Additional file 1, Table S2). HFD-treated 3 × Tg mice also exhibited increased platelet production (thrombocytosis) and MPV that could be partially attributed to elevated IL-6 and TPO, which induce MK differentiation into platelets. Large-sized platelets have been shown to be more active than small platelets and can produce more thromboxane A2 resulting in sustained platelet activation and aggregation [57]. Hence, large platelets are associated with a poor outcome in acute myocardial infarction and ischemic stroke [58, 59]. Collectively, our data suggest that the blood of HFD-treated 3 × Tg mice is in a prothrombotic state, which increases the risk of cerebral circulatory deficits resulting in memory deficits, as observed in the current study.

Integrin a_IIb_β_3_ (GPIIb/IIIa) is a heterodimeric receptor of the integrin family expressed at high density (50,000–80,000 copies/cell) on the platelet membrane [60]. In resting platelets, a_IIb_β_3_ exists in a low-affinity state and does not bind its ligands, such as fibrinogen, vWF, fibronectin, and monomeric Aβ40. However, sustained platelet activation may result in the increased expression of a_IIb_β_3_ by alpha granules and exposure of the binding site(s) of a_IIb_β_3_ for a variety of ligands, including Aβ40. Previously, Donner et al. have demonstrated that Aβ40 could bind to a_IIb_β_3_ through its RHDS sequence, which causes integrin outside-in signaling and downstream activation of Syk and PLCr2, ultimately promoting the release of the chaperone clusterin and ADP from alpha and dense granules of activated platelets, respectively [44]. The release of clusterin facilitates the conversion of soluble Aβ40 into fibrillar Aβ aggregates [44]. Consistent with this finding, we report that the expression of integrin a_IIb_β_3_ and clusterin were significantly increased in platelets of HFD-treated 3 × Tg mice. These platelets actively induced the conversion of soluble Aβ40 into fibrillar Aβ aggregates.

At 9 months and older, HFD-treated 3 × Tg had platelet-associated fibrillar Aβ clots resulting in occlusion at sites of Aβ deposits in the cerebral vessels. It is conceivable that the blocked blood vessels may further promote atherosclerosis-induced hypoperfusion, hypoxia, and other vascular dysfunction, consistent with the observed cerebral vascular leakage and increased tau pathology and loss of neurons in HFD-treated 3 × Tg mice. Given our data that the contribution of atherosclerosis to AD-related pathology is at least in part via facilitating the formation of platelet-associated fibrillar Aβ aggregates, a drug that could directly dissolve platelet micro-clots would in theory normalize any platelet-Aβ clots formed in the brain. This would improve cerebral blood flow, and both neuronal function and survival. In this study, we describe a novel therapeutic strategy for clearance of preexisting platelet-Aβ clots with scFv Ab (A11) that specifically fragments activated platelet by targeting platelet GPIIIa49-66. In the presence of A11, platelet thrombi were disaggregated, thus preventing the transformation of soluble Aβ40 into fibrillar Aβ on the surface of platelet thrombi in vitro. 3 × Tg mice with atherosclerosis being treated with A11 for a period of 3 months demonstrated reduced vascular permeability, in the absence of any bleeding risk. This approach, perhaps in conjunction with other synergistic strategies, could have potential therapeutic benefits for the treatment of AD.

### Limitations

In this study, we found that the expressions of integrin a_IIb_β_3_ and clusterin were significantly increased in platelets of HFD-treated 3 × Tg mice. These murine platelets actively induced the conversion of soluble Aβ40 into fibrillar Aβ aggregates in vitro. Further investigations with human platelets from AD patients with atherosclerosis are needed to validate these results. In addition, the current data have established the concept of developing a different approach to combat AD by lysing platelet micro-clots. Although A11 reduced the formation of platelet-associated fibrillar Aβ aggregates in vitro and appeared to improve vascular permeability, it demonstrated little effect on mouse cognitive ability. Thus, A11 treatment needs to be further evaluated in a larger cohort with optimization of the protocol, in future studies.

## Conclusion

In summary, our studies suggest that the major contribution of atherosclerosis to AD pathology is via its effects on blood coagulation, increased number and activation of platelets, and the formation of platelet-mediated Aβ clots. The latter compromises cerebral blood flow, producing neuronal loss, and enhances tau-related pathology, resulting in cognitive decline. Our findings also suggest that clearance of preexisting platelet micro-clots is a potential therapeutic approach for AD treatment.

## Supplementary Information

**Additional file 1: Table S1.** Antibodies used in this study. **TableS2.** Serum differentially expressed proteins between high-fat diet-treated (H) and normal-chow-treated (N) 3 × Tg mice. **Figure S1.** Gene ontology (GO) term and pathway analyses of significantly changed proteins by Metascape revealed enrichment of proteins of several pathways related to complement and coagulation cascades, blood coagulation, platelet degranulation, and cell-substrate adhesion. **Figure S2.** Initial CAA lesions in HFD-treated 3 × Tg mice. **Figure S3.** Safety of A11 injection on other organs..

## Data Availability

Requests for resources, reagents, and further information will be made available from the lead corresponding author (Wei Zhang) on reasonable request.

## Abbreviations

3 × Tg: Triple transgenic
AD: Alzheimer’s disease
Aβ: β-amyloid
HFD: High-fat diet
CAA: Cerebral amyloid angiopathy
CBF: Cerebral blood flow
BBB: Blood-brain barrier
NFT: Neurofibrillary tangles
APP: Amyloid precursor protein
scFv: Single-chain variable fragment
Ab: Antibody
WT: Wild-type
TC: Total cholesterol
TAs: Thoracic-abdominal aortas
RT: Room temperature
PRP: Platelet-rich plasma
BSA: Bovine serum albumin
PFA: Paraformaldehyde
TBS: Tris-buffered saline
MPV: Mean platelet volume
*q*RT-PCR: Quantitative real-time RT-PCR
TPO: Thrombopoietin
PBS: Phosphate-buffered saline
ROS: Reactive oxygen species
GSH: Glutathione
ET: Endostatin
SDS–PAGE: Sodium dodecyl sulfate polyacrylamide gel electrophoresis
PVDF: Polyvinylidene difluoride
HRP: Horseradish peroxidase
ECL: Enhanced chemiluminescence
HE: Hematoxylin and eosin
GFAP: Glial fibrillary acidic protein
i.p.: Intraperitoneally injection
MS: Mass spectrometry
GO: Gene ontology
vWF: Von Willebrand Factor
C: Complement
ITIH1: Inter-alpha-trypsin inhibitor heavy chain H1
GSN: Gelsolin
MKs: Megakaryocytes
IL-6R: IL-6 receptor
IHC: Immunohistochemistry
ECs: Endothelial cells
PY: Pyramidal layer
DG: Dentate gyrus
ADP: Adenosine diphosphate
RBC: Red blood cell
LBC: Lymphocyte
CLU: Clusterin
