# Supplementary material for: High-fat diet-induced atherosclerosis promotes neurodegeneration in the triple transgenic (3 × Tg) mouse model of Alzheimer’s disease associated with chronic platelet activation
Source: Alzheimers Res Ther. 2021 Aug 28;13:144. doi: 10.1186/s13195-021-00890-9 (PMC8403418; doi:10.1186/s13195-021-00890-9)
Supplement: Supplementary file 1 — Additional file 1: Table S1. Antibodies used in this study. TableS2. Serum differentially expressed proteins between high-fat diet-treated (H) and normal-chow-treated (N) 3 × Tg mice. Figure S1. Gene ontology (GO) term and pathway analyses of significantly changed proteins by Metascape revealed enrichment of proteins of several pathways related to complement and coagulation cascades, blood coagulation, platelet degranulation, and cell-substrate adhesion. Figure S2. Initial CAA lesions in HFD-treated 3 × Tg mice. Figure S3. Safety of A11 injection on other organs.. [file 13195_2021_890_MOESM1_ESM.docx]

**Supplemental Information**

**High-Fat Diet-induced atherosclerosis promotes neurodegeneration in the triple transgenic (3 × Tg)** **mouse model of Alzheimer's disease associated with chronic platelet activation**

Min Wang^1#^, Junyan Lv^1#^, Xiaoshan Huang^1^, Thomas Wisniewski^2*^,Wei Zhang^1*^

^1^Key Laboratory of Brain Functional Genomics (Ministry of Education and Shanghai), School of Life Sciences, East China Normal University, Shanghai, China;

^2^Center for Cognitive Neurology and Departments of Neurology, Pathology and Psychiatry, New York University School of Medicine, New York, NY 10016, USA

#These authors contribute equally to this work.

*Correspondence: Dr. Wei Zhang, East China Normal University, 3663 North Zhongshan Road, Shanghai 200062, China; E-mail address: [wzhang@sat.ecnu.edu.cn](mailto:wzhang@sat.ecnu.edu.cn); Tel: +86 21 62233980; Fax: +86 21 62233980.

*Correspondence: Dr. Thomas Wisniewski, Center for Cognitive Neurology and Departments of Neurology, Pathology and Psychiatry, NYU School of Medicine, Science Building, Rm1017, 435 East 30^th^ Street, New York, NY, 10016, USA. E-mail address: [Thomas.wisniewski@nyulangone.org](mailto:Thomas.wisniewski@nyulangone.org).

***Methods***

*Proteomic analysis*

Mouse serum proteins (100 μg) were reduced by 10 mM dithiothreitol (DTT) at 37°C for 1 h, and alkylated with 50 mM iodoacetamide (IAA) in the dark at room temperature for 10 min to block the cysteine residues. The samples were then desalted and buffer-changed three times with 100 μl 0.5M triethylammonium bicarbonate (TEAB) by using ultracentrifugal filter devices. The proteins were then digested with sequencing grade trypsin (Promega) and fractionated with high PH reversed phase chromatography. The data-independent acquisition (DIA) analysis was performed on an Orbitrap Fusion LUMOS mass spectrometer (Thermo Fisher Scientific) connected to an Easy-nLC 1200 via an Easy Spray (Thermo Fisher Scientific). The DIA raw files were analyzed in Spectronaut X (Biognosys, Schlieren, Switzerland). Pathway enrichment analysis was performed with MetaScape (http:// metascape.org/).

***Results***

**Supplemental Figures**

**
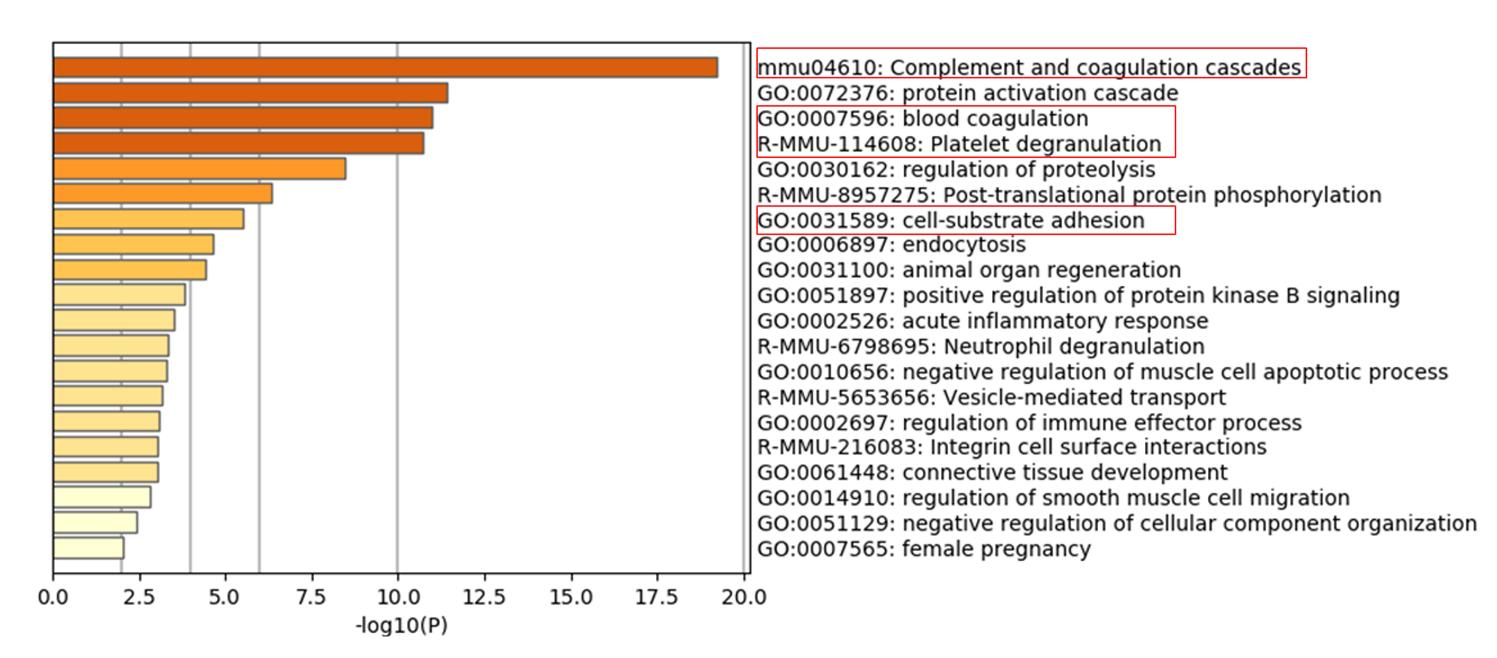
**

**Figure S1** Gene ontology (GO) term and pathway analyses of significantly changed proteins by Metascape revealed enrichment of proteins of several pathways related to complement and coagulation cascades, blood coagulation, platelet degranulation, and cell-substrate adhesion.


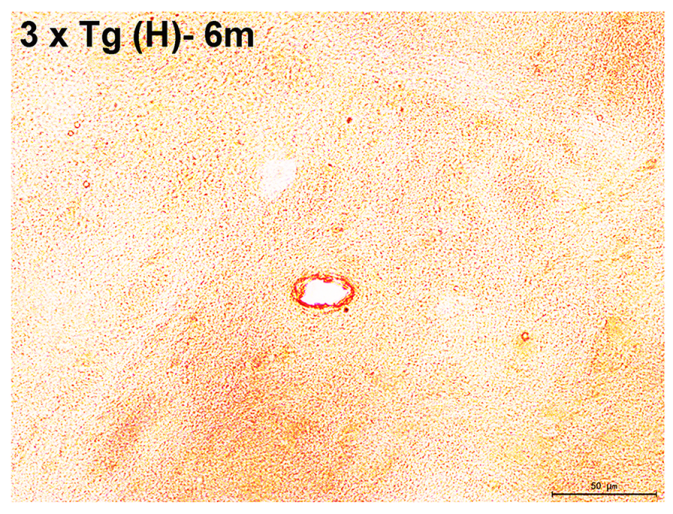


**Figure S2** Representative images of Congo red staining-positive CAA lesions in cerebrovascular vessels of HFD-treated 3 × Tg mice at 6 months. Scale bar, 50 μm. **
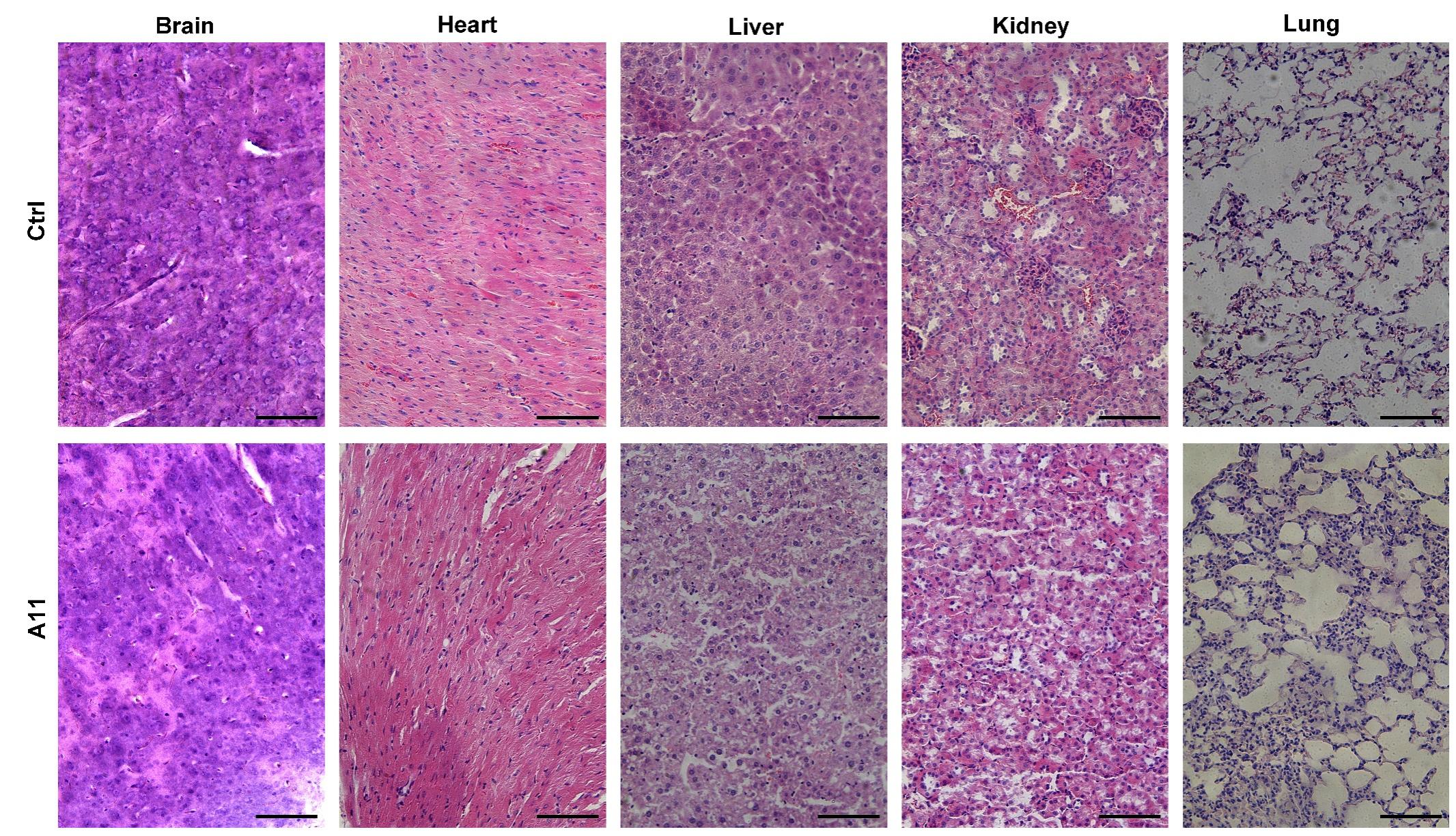
**

**Figure S3** Safety of A11 injection on other organs. No significant pathological changes were observed in the brain, heart, liver, kidney, or lung by histologic examination, suggesting that the treatment was apparently harmless to mice.

**Supplemental Tables**

**Table S1** Antibodies used in this study

| Name | Catalog number | Company | Application  (dilution) |
| --- | --- | --- | --- |
| Anti-GPIbα | M042-0 | emfret analytis | IHC/ICC (1:100) |
| Anti-Aβ (1-16) | 802901 | Biolegend | IHC/ICC (1:800) |
| Anti-GFAP | 16825-1-AP | Proteintech | IHC (1:300) |
| DAPI | / | MP Biomedicals | IHC (10μg/ml) |
| Anti-CD41 | ab-95725 | Abcam | FCM (0.12 μg for 10^5^ cell) |
| Anti-Integrin beta 3 | Ab119992 | Abcam | WB (1:1000) |
| Alexa Fluor 488 Donkey Anti-Rabbit IgG | 705-545-147 | Jackson Immuno Research | IHC/ICC (1:200) |
| CY^TM^3 Affinity Goat Anti-Rat IgG | 112-165-003 | Jackson Immuno Research | IHC/ICC (1:200) |
| Tau | ab32057 | Abcam | WB (1:5000) |
| p-Tau | ab109390 | Abcam | WB (1:10000)/IHC (1:100) |
| NeuN | ab177487 | Abcam | IHC (1:500) |

**Table S2** Serum differentially expressed proteins between high-fat diet-treated (H) and normal-chow-treated (N) 3 × Tg mice (n = 5/group)

| **No.** | **Protein Name** | **Genes** | **Uniprot IDs** | **Log2Ratio (H:N)** | **Q value** | **Involved Pathways** | **Pathophysiological role and association with AD** |
| --- | --- | --- | --- | --- | --- | --- | --- |
| 1 | SERPINA1 | Alpha-1-antitrypsin | Q00898 | 2.29 | 0.02474 | Acute inflammatory response | SERPINA1 has been found to be associated with the inflammatory process and localized in neurofibrillary tangles and senile plaques^[4]^. SERPINA1 has been found to be significantly increased in AD blood ^[5,6]^. |
| 2 | Von Willebrand Factor | Vwf | E9QPU1 | 2.18 | 0.00737 | Coagulation and platelet degranulation | vWF is a large multimeric glycoprotein that could be released by platelet alpha granules upon platelet activation ^[1]^. As an adhesive ligands, vWF initiates platelet adhesion to the injured vascular wall by binding to glycoprotein GPIba on platelets, promoting platelet aggregation and activation of the procoagulative function leading to diminished blood circulation to vital organs. It has been shown that vWF was abnormally high in AD patients ^[2]^. |
| 3 | Complement C5 | C5 | P06684 | 1.54 | 0.01753 | Complement cascades | Complement activation is a pivotal process of neuroinflammation that has been reported in AD cohorts ^[3]^. |
| 4 | Mannan-binding lectin serine protease 2 | Masp2 | Q91WP0 | 1.52 | 0.00767 | Coagulation cascades |  |
| 5 | Inter-alpha-trypsin inhibitor heavy chain H2 | Itih2 | Q61703 | 1.54 | 0.007602 |  | Hyaluronan metabolism, inflammation |
| 6 | Serum albumin | Alb | P07724 | 1.51 | 1.41E-07 | Coagulation and hemostasis | Neuroinflammation |
| 7 | Coagulation factor X | F10 | Q3U3V1 | 1.49 | 0.04495 | Coagulation cascades |  |
| 8 | Complement Factor I | Cfi | Q61129 | 1.44 | 6.17E-05 | Complement cascades | Neuroinflammation |
| 9 | Fibulin-1 | Fbln1 | Q08879 | 1.39 | 0.016934 | Coagulation and hemostasis | FBLN1 is a kind of extracellular matrix (ECM) protein, and was found to be increased in AD serum ^[9]^. FBLN1 plays an important role in maintaining hemostasis and thrombosis due to its ability to bind with fibrinogen and to be incorporated into clots ^[10]^. |
| 10 | Complement C3 | C3 | P01027 | 1.33 | 1.40E-05 | Complement cascades and platelet degranulation | The generation of C3 is closely associated with platelet degranulation upon activation and is involved in neuroinflammation |
| 11 | Vitronectin | Vtn | P29788 | 1.30 | 0.01082 | Blood coagulation | Platelet adhesion and activation |
| 12 | Plasminogen | Plg | P20918 | 1.26 | 0.002346 | Coagulation cascades |  |
| 13 | Talin-1 | Tln1 | P26039 | 1.18 | 1.40E-05 | Blood coagulation | Platelet activation |
| 14 | Fibronectin | Fn1 | A0A087WSN6 | 1.17 | 3.43E-07 | Blood coagulation | Acute phase, angiogenesis, platelet activation, cell adhesion and migration |
| 15 | Ceruloplasmin | CP | Q61147 | 1.14 | 0.044953 | Acute inflammatory response | Iron homeostasis, acute phase |
| 16 | Prothrombin | F2 | P19221 | 1.04 | 2.82E-09 | Coagulation cascades | Coagulation and hemostasis |
| 17 | Gelsolin | Gsn | P13020 | 0.98 | 0.049738 |  | GSN is a cytoskeletal protein and is present both intracellularly and extracellularly ^[11]^. Previous studies have identified the anti-amyloidogenic role of GSN in AD patients and showed that both plasma and cytosolic GSNs bound to Aβ to inhibit the fibrillization of Aβ ^[11]^. |
| 18 | Afamin | Afm | O89020 | 0.96 | 1.28E-06 |  | Transport of vitamin E |
| 19 | Antithrombin-III | Serpinc1 | P32261 | 0.85 | 0.022803 | Coagulation and hemostasis |  |
| 20 | Inter-alpha-trypsin inhibitor heavy chain H1 | Itih1 | Q61702 | 0.85 | 0.007672 | Acute inflammatory response | ITIH1 and ITIH2 are acute-phase protein. They are the heavy chains of a serine protease inhibitor, which may act as carrier of hyaluronan in serum. Previous studies showed that ITIH1 and ITIH2 levels were lowered in AD blood ^[8]^. However, their exact roles in AD remain unclear. |
| 21 | Plasma kallikrein | Klkb1 | P26262 | 0.81 | 0.000834 | Coagulation cascades |  |
| 22 | Complement C4-B | C4b | P01029 | 0.74 | 0.044953 | Complement cascades | Neuroinflammation |
| 23 | Apolipoprotein A-I | APOA1 | Q00623 | 0.66 | 2.34E-07 |  | APOA1 is a major component of the high density lipoproteins that modulates the toxic effect of amyloid at several levels, including a capacity to prevent β-sheet organization and physically inhibit amyloid aggregation ^[7]^. |
| 24 | Thrombospondin-1 | Thbs1 | Q80YQ1 | 0.66 | 5.21E-09 | Coagulation and hemostasis |  |
| 25 | Alpha-2-macroglobulin-P | A2m | Q6GQT1 | 0.59 | 0.006774 | Coagulation cascades |  |

**Reference (From Table S2)**

1. Dmitrieva NI, Burg MB. Secretion of von Willebrand factor by endothelial cells links sodium to hypercoagulability and thrombosis. Proc Natl Acad Sci U S A. 2014;111:6485-90.

2. O'Bryant SE, Xiao G, Barber R, Reisch J, Doody R, Fairchild T, Adams P, Waring S, Diaz-Arrastia R; Texas Alzheimer's Research Consortium. A serum protein-based algorithm for the detection of Alzheimer disease. Arch Neurol. 2010;67:1077-81.

3. Wang Y, Hancock AM, Bradner J, Chung KA, Quinn JF, Peskind ER, Galasko D, Jankovic J, Zabetian CP, Kim HM, Leverenz JB, Montine TJ, Ginghina C, Edwards KL, Snapinn KW, Goldstein DS, Shi M, Zhang J. Complement 3 and factor h in human cerebrospinal fluid in Parkinson's disease, Alzheimer's disease, and multiple-system atrophy. Am J Pathol. 2011;178:1509-16.

4. Gollin PA, Kalaria RN, Eikelenboom P, Rozemuller A, Perry G. Alpha 1-antitrypsin and alpha 1-antichymotrypsin are in the lesions of Alzheimer's disease. Neuroreport. 1992;3:201-3.

5. Liao PC, Yu L, Kuo CC, Lin C, Kuo YM. Proteomics analysis of plasma for potential biomarkers in the diagnosis of Alzheimer's disease. Proteomics Clin Appl. 2007;1:506-12.

6. Doecke JD, Laws SM, Faux NG, Wilson W, Burnham SC, Lam CP, Mondal A, Bedo J, Bush AI, Brown B, De Ruyck K, Ellis KA, Fowler C, Gupta VB, Head R, Macaulay SL, Pertile K, Rowe CC, Rembach A, Rodrigues M, Rumble R, Szoeke C, Taddei K, Taddei T, Trounson B, Ames D, Masters CL, Martins RN; Alzheimer's Disease Neuroimaging Initiative; Australian Imaging Biomarker and Lifestyle Research Group. Blood-based protein biomarkers for diagnosis of Alzheimer disease. Arch Neurol. 2012;69:1318-25.

7. Roher AE, Maarouf CL, Sue LI, Hu Y, Wilson J, Beach TG. Proteomics-derived cerebrospinal fluid markers of autopsy-confirmed Alzheimer's disease. Biomarkers. 2009;14:493-501.

8. Güntert A, Campbell J, Saleem M, O'Brien DP, Thompson AJ, Byers HL, Ward MA, Lovestone S. Plasma gelsolin is decreased and correlates with rate of decline in Alzheimer's disease. J Alzheimers Dis. 2010;21:585-96.

9. Shen L, Liao L, Chen C, Guo Y, Song D, Wang Y, Chen Y, Zhang K, Ying M, Li S, Liu Q, Ni J. Proteomics Analysis of Blood Serums from Alzheimer's Disease Patients Using iTRAQ Labeling Technology. J Alzheimers Dis. 2017;56:361-378.

10. Timpl R, Sasaki T, Kostka G, Chu ML. Fibulins: a versatile family of extracellular matrix proteins. Nat Rev Mol Cell Biol. 2003;4:479-89.

11. Yang W, Chauhan A, Mehta S, Mehta P, Gu F, Chauhan V. Trichostatin A increases the levels of plasma gelsolin and amyloid beta-protein in a transgenic mouse model of Alzheimer's disease. Life Sci. 2014;99:31-6.
